# Systemic Sclerosis-Associated Pulmonary Arterial Hypertension: From Bedside to Bench and Back Again

**DOI:** 10.3390/ijms25094728

**Published:** 2024-04-26

**Authors:** Milan Bahi, Christine Li, Gaochan Wang, Benjamin D. Korman

**Affiliations:** Division of Allergy, Immunology, and Rheumatology, University of Rochester Medical Center, 601 Elmwood Ave, Box 695, Rochester, NY 14642, USA; milanbahi02@gmail.com (M.B.);

**Keywords:** systemic sclerosis, scleroderma, pulmonary hypertension, pulmonary arterial hypertension, biomarkers, molecular pathogenesis, therapeutics

## Abstract

Systemic sclerosis (SSc) is a heterogeneous disease characterized by autoimmunity, vasculopathy, and fibrosis which affects the skin and internal organs. One key aspect of SSc vasculopathy is pulmonary arterial hypertension (SSc-PAH) which represents a leading cause of morbidity and mortality in patients with SSc. The pathogenesis of pulmonary hypertension is complex, with multiple vascular cell types, inflammation, and intracellular signaling pathways contributing to vascular pathology and remodeling. In this review, we focus on shared molecular features of pulmonary hypertension and those which make SSc-PAH a unique entity. We highlight advances in the understanding of the clinical and translational science pertinent to this disease. We first review clinical presentations and phenotypes, pathology, and novel biomarkers, and then highlight relevant animal models, key cellular and molecular pathways in pathogenesis, and explore emerging treatment strategies in SSc-PAH.

## 1. Introduction

Systemic sclerosis (SSc) is a heterogeneous autoimmune disorder characterized by chronic inflammation, endothelial dysfunction, and fibrosis of the skin and internal organs [1]. The prevalence of SSc worldwide is approximately 176 cases per one million population with the annual incidence of 14 cases per one million person-year [2]. SSc affects women more frequently than men with a 3–4:1 female:male ratio; however, men with SSc experience more severe outcomes [1].

While fibrosis is generally considered the hallmark of SSc, vascular disease plays a significant role in pathogenesis, and vascular pathology occurs early and represents a key driver of morbidity. Pulmonary hypertension (PH) is a progressive devastating disease affecting small and medium pulmonary arteries, causing pulmonary vascular remodeling which can occur due to multiple etiologies. This process leads to right ventricular dysfunction, heart failure, and ultimately death if untreated [3,4]. Pulmonary arterial hypertension (PAH) is reported to occur in 8–12% of SSc patients (SSc-PAH) and represents a leading cause of mortality in individuals with SSc [5]. Recent studies have shown that the combination of early detection of disease resulting from advances in screening guideline and PAH specific drugs improved the survival among SSc-PAH patients [6].

Though data specific to SSc-PAH is sometimes limited, elucidation of molecular mechanisms underlying PAH has significantly improved, and better understanding of disease pathogenesis has contributed to the identification of new targets for drug development. In this review, in addition to reviewing clinical features, we will emphasize the pathology of PAH, phenotypes and biomarkers of SSc-PAH, cellular and molecular mechanisms underlying SSc-PAH, relevant animal models, and treatment for SSc-PAH with a focus on the basic understanding of disease biology influences diagnosis, prognosis, and treatment strategies.

While not a systematic review, methodologically we reviewed the literature using Pubmed/MEDLINE searches for the primary medical literature between 2000 and 2024 which included the terms “SSc-PAH”, “systemic sclerosis associated pulmonary arterial hypertension”, “scleroderma associated pulmonary arterial hypertension”, “systemic sclerosis”, “pulmonary arterial hypertension”, and “pulmonary hypertension”, and then added additional filters including “classification”, “diagnosis”, “pathology”, “histology”, “animal model”, “model”, “pathway”, “signaling”, “molecular”, “cellular”, “treatment”, “digital ulceration”, and “clinical trial” for the review of the literature in each individual section. To ensure broad coverage of the literature, we allowed inclusion of one to two review articles per section with a focus on recent (since 2018) or seminal reviews of highly relevant topics, and later added selected older references which were the first studies to describe important relevant findings. Three individuals (M.B., C.L., and G.W.) performed the searches between December 2023 and March 2024 and selected 193 articles from over 2900 articles in the initial search filters. Articles were selected if they were deemed to be specific to SSc-PAH or highlighted important common pathobiology or clinical aspects of PH. When multiple references addressed a given aspect of the topic, the most recent literature was selected for inclusion with the majority of references (69%) representing the literature from the last 10 years.

## 2. Clinical Aspects and Definitions

### 2.1. Systemic Sclerosis

Systemic sclerosis (SSc) is a multisystem autoimmune connective tissue disease characterized by microvascular injury, dysregulation of adaptive and innate immunity, and fibrosis of the skin and internal organs. Common clinical features of SSc include Raynaud’s phenomenon, skin thickening, calcinosis, telangiectasias, gastroesophageal reflux disease, GI dysmotility, arthritis, interstitial lung disease, and pulmonary hypertension [7]. Among these, Raynaud’s phenomenon, a vasospasm of the digits, is an early finding which typically occurs before fibrotic features, suggesting that vascular disease is an early manifestation and plays a role in early pathogenesis. Pulmonary hypertension is usually a later manifestation of SSc and typically presents 10–15 years after diagnosis.

### 2.2. Pulmonary Hypertension—WHO Classification

Pulmonary hypertension (PH) is a broad term used to describe a group of conditions with the common characteristic of an increase in pulmonary arterial pressure. The World Health Organization (WHO) currently defines pre-capillary PH as a mean pulmonary arterial pressure (mPAP) > 20 mmHg, pulmonary arterial wedge pressure (PAWP) ≤ 15 mmHg, and pulmonary vascular resistance PVR ≥ 3 Woods Units [8]. This definition changed in 2018 (prior definitions used mPAP > 25 mmHg) to identify patients with earlier disease.

Pulmonary hypertension can be classified into five main categories: pulmonary arterial hypertension (PAH, Group 1), PH due to left heart disease (PH-LHD, Group 2), PH due to lung diseases and/or hypoxia (PH-lung, Group 3), PH due to pulmonary artery obstructions (CTEPH, Group 4), and PH with unclear and/or multifactorial mechanisms (Group 5) [8]. Interestingly, patients with SSc can have features of all of these subtypes of PH.

Group 1 PH represents pulmonary arterial hypertension (PAH), a primary disease caused by arterial dysfunction including narrowing and stiffening of the pulmonary arteries [9]. Characteristics of this category include progressive pulmonary arterial vasculopathy, increased pulmonary vascular resistance (PVR) and increased right ventricular (RV) afterload. The increase in RV afterload is associated with RV dysfunction and RV failure [9]. The most common form of PAH is idiopathic PAH (IPAH), followed by connective-tissue-disease-associated PAH (CTD-PAH), genetic forms of PAH, drug-induced PAH, and PAH secondary to HIV, portal hypertension, or congenital heart disease. Amongst the connective tissue disease associated subtype, SSc, systemic lupus erythematosus, mixed connective tissue disease, Sjogren’s syndrome, and rheumatoid arthritis can all cause PAH although SSc represents about 75% of CTD-PAH cases.

WHO Group 2 represents PH due to left heart disease (PH-LHD), and occurs in the setting of high left atrial pressure due to cardiac disease, leads to engorgement of the pulmonary vein, and primarily represents pulmonary venous hypertension [9]. The pathophysiology of Group 2 PH is thought to result from increased wall stress due to increased left atrial pressures, decreased shear stress in the pulmonary vascular bed, and endothelial dysfunction [9]. Because the primary pathology leading to post-capillary PH is elevation in left atrial or ventricular filling pressures, this can be a feature of heart failure of any etiology including mitral stenosis, cardiomyopathy, or LV diastolic dysfunction. While ischemic and non-ischemic cardiomyopathy represent the majority group 2 PH, in SSc, there is a high prevalence of heart failure with preserved ejection fraction (HFpEF), and this represents an important and under-appreciated form of PH is SSc [10].

Group 3 is PH due to lung diseases and/or hypoxia (PH-lung). This occurs when patients with lung disease, hypoxia, or a combination of both, leading to low oxygen levels in the lungs. Chronic obstructive pulmonary disease (COPD), interstitial lung disease (ILD) and obstructive sleep apnea (OSA), cystic fibrosis, and high altitude exposure are among the most likely lung diseases to predispose to this presentation [9]. These illnesses cause a loss of blood vessel density and therefore make it more challenging to accommodate higher cardiac output, leading to pulmonary pressure increase. Moreover, lung disease leads to periods of continuous or intermittent hypoxia which can lead to constriction of the pulmonary vessels. In SSc, registries have approximated that up to 65% of patients have or will develop radiographic evidence of ILD in the course of their disease [11]. While only a minority of these cases will be clinically significant and progressive, patients with advanced SSc-ILD often develop concomitant PH consistent with Group 3 disease, which portends a poor prognosis [12].

Group 4 PH is chronic thromboembolic pulmonary hypertension (CTEPH), which is PH due to pulmonary artery obstruction. This occurs when the lungs are unable to resolve a thrombus causing scar tissue in the pulmonary vessels which obstructs blood flow [13]. Causes of a hypercoagulable state which predispose to pulmonary emboli are the primary etiology Group 4 PH. In SSc, while CTEPH is rare, patients do have increased prevalence of antiphospholipid antibodies which can predispose to pulmonary emboli.

Group 5 is PH with unclear and/or multifactorial mechanisms. Some of these diseases include chronic hemolytic anemias, splenectomy, and metabolic disorders. These diseases all show a correlation with higher PAP, but the mechanism through which this occurs is generally not well understood [10].

Given the multiple potential presentations of PH in SSc, patients diagnosed with PH of any etiology should undergo workup to evaluate for concomitant interstitial lung disease, cardiac disease including systolic and diastolic heart failure and valvular heart disease, and a hypercoagulable state. While PAH remains the most common etiology, patients may present with components of multiple types of PH and treatment may be tailored to the various etiologies.

### 2.3. SSc PH and SSc-PAH

SSc-PH is estimated to affect 15–18% of patients with the most prevalent form being SSc-PAH [5], which affects 6–9% SSc patients and has traditionally been deemed the form with the highest mortality [5,14]. The prognosis of SSc-PAH is poor and has been reported as low as a 3-year survival of 30% [15]. However, recent studies using more modern treatment algorithms report better outcomes with a 3-year survival of 62% [16], and report that transplant-free survival has improved significantly in Group 1 PH over the last decade, likely secondary to earlier detection and better therapeutic management. Despite these advances, SSc-PAH patients continue to have worse outcomes than patients with other forms of PAH, likely due to multiple medical comorbidities. In contrast to PAH, SSc patients with Group 2 or 3 PH continue to have a poor prognosis [6].

## 3. Pathology of PAH

PAH has a few common pathological features. These include remodeling of the three layers of the distal pulmonary vasculature, extension of the smooth muscle cell layer to typically non-vascularized distal capillaries, and in situ thrombosis involving small muscular arteries [17]. All of these features result in luminal narrowing or complete obliteration of small vessels, medial hypertrophy, intimal fibrosis, adventitial thickening, and thrombosis [18] and can result in plexiform lesions in severe disease [17,19]. Although the exact mechanisms of more severe remodeling in PAH has not been determined, endothelial dysfunction, abnormal shear stress, and inflammation have been implicated [17].

### SSc-PAH vs. IPAH

**Figure 1:**
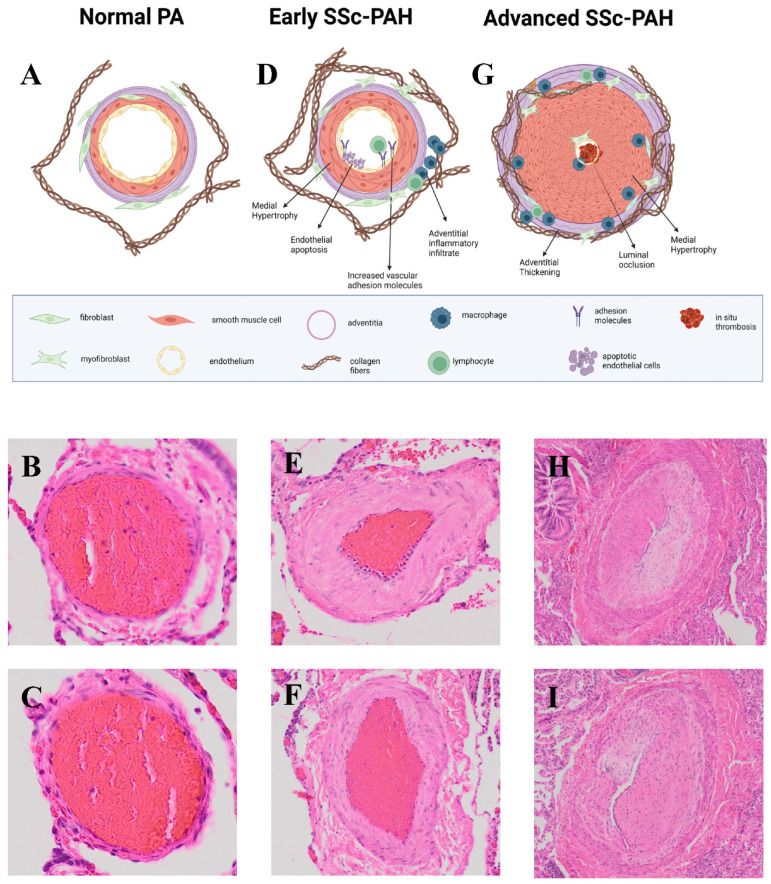
Cellular mechanisms and histology of SSc-PAH. (**A**) Schematic representing a cross-section of pulmonary artery from a control. Created with BioRender.com. (**B**,**C**) Representative images of pulmonary artery histology (H&E staining, 20×) from two control individuals with no signs of PAH. Note the open lumen, thin media, and adventitia without perivascular inflammation. (**D**) Diagram of early SSc-PAH. Note the increased medial hypertrophy, luminal narrowing, endothelial apoptosis presence of vascular adhesion molecules, and adventitial inflammatory infiltrates including macrophages and lymphocytes. (**E**,**F**) Partially occluded pulmonary arterial vessels from patients with SSc-PAH. Note increased thickness of the media causing luminal narrowing and adventitial proliferation. (**G**) Schematic of advanced SSc-PAH. Note luminal occlusion due to adventitial thickening, medial hypertrophy, and the presence of in situ thrombosis, as well as the presence of macrophages, lymphocytes, and myofibroblasts now infiltrating into the adventitial and medial layers. (**H**,**I**) Histology demonstrating complete luminal occlusion in severe SSc-PAH. Note extensive thickening of the adventitial and medial layers causing luminal obliteration as well as significant adventitial inflammatory infiltrate.

The pathology of SSc-PAH is similar to PAH as a whole with pulmonary vascular remodeling of the medium and small arteries and pulmonary arterial obstruction [20]. The pulmonary vascular remodeling of SSc-PAH is characterized by medial hypertrophy, intimal hyperplasia, and pulmonary adventitial thickening with inflammatory infiltrates (Figure 1). More rarely, biopsies may show thrombosis in situ, pulmonary veno-occlusive disease, and plexiform arteriopathy [21,22].

There are a number of studies comparing SSc-PAH to IPAH in its pathology. For example, one study found that the two entities were similar in their presentation of pulmonary arterial/arteriolar intimal fibrosis as well as the fibrosis of pulmonary veins/venules [22]. However, most IPAH patients showed evidence of plexogenic arteriopathy while none of the SSc-PAH patients did [22]. Another difference was that a significant portion of SSc-PAH patients demonstrated fibrosis of veins/venules associated with capillary congestion similar to pulmonary veno-occlusive disease [22]. Another study found a larger proportion of SSc-PAH biopsies showed marked muscular artery intimal fibrosis and pulmonary venous lesions compared to IPAH [21,23]. The differences shown in small vessel intimal fibrosis may provide insight into the differences in pathogenetic mechanisms between SSc-PAH and IPAH groups [22].

There were also studies that looked at right ventricular function in SSc-PAH patients vs. IPAH patients. Two pathologic investigations saw worse right ventricular contractility and coupling of RV contractility in SSc-PAH patients compared to IPAH patients [24,25]. There are conflicting conclusions, however, in studies on the extent of interstitial fibrosis in the right ventricle of SSc-PAH vs. IPAH patients, with one study finding no difference in the extent of interstitial fibrosis in the RVs while another observed increased RV interstitial fibrosis [24,26]. Additionally, sarcomere function was significantly lower in SSc-PAH than in IPAH [24].

While similar pathologically, the distinctions in pathology between SSc-PAH vs. IPAH patients indicate that the two conditions are separate entities. While lung biopsy is being performed less frequently and gross histology may not be sufficient to ascertain why these differences occur, the advent of technologies such as spatial transcriptomics, which have begun to offer insights in SSc skin fibrotic and vascular disease [27,28], may allow for further identification of biologic differences which make SSc-PAH more likely to include pulmonary veins, less likely to form plexogenic lesions, and more likely to lead to RV dysfunction.

## 4. PAH Genetics

Heritable genetic forms of pulmonary arterial hypertension, although relatively rare, are important because they give insight into disease pathogenesis. Mendelian inheritance of PAH has been reported primarily in patients carrying mutations in the *BMPR2, ALK1*, and *ENG* genes, with 70–80% of familial PAH and 10–20% of IPAH cases demonstrating mutations in *BMPR2* [29]. *BMPR2* loss-of-function mutations promote pro-proliferative signaling, resulting in characteristic PAH vasculopathy [30]. Interestingly, patients with IPAH without mutations demonstrate reduced BMPR2 expression in lung tissue [31] suggesting that this may be a common downstream pathway. Moreover, mutations in the effectors of TGF-β superfamily receptors have commonly been described in patients with familial PAH, IPAH, and hereditary hemorrhagic telangiectasia [32]. More recently, several rare variants have been identified in additional genes including *SMAD9*, *SMAD1*, *CAV1*, *KCNK3*, *TBX4*, *ATP13A3 SOX17*, *AQP1*, *GDF2*, and *EIF2AK4* [33]. Whole-exome sequencing (WES) has suggested that key pathways including cytoskeletal function and the Wnt signaling are preferentially involved in cases with rare variants [34]. 

Multiple genes, mostly MHC variants, and genes in immune pathways have been implicated in SSc pathogenesis by GWAS and exome sequencing [35]. Unfortunately, most of these studies have not had enough patients with rare phenotypes like PAH to definitively assess whether variants associated with overall disease risk are also associated with PAH or to investigate this phenotype exclusively. Unlike heritable PAH, there is no clear mendelian gene associations with SSc-PAH and studies of SSc-PAH have failed to identify mutations reported in hereditary and IPAH. A cohort study in SSc-PAH patients concluded that there was a lack of association between TGF-β receptor polymorphisms seen in IPAH and SSc-PAH [36]. However, certain genetic dispositions may still increase disease susceptibility. Studies have shown that polymorphisms in *MIF*, *TLR2*, *UPAR*, *KCNK5*, and *HLA-B35* may predispose to PAH onset in SSc patients [37]. 

## 5. Biomarkers

Because monitoring of hemodynamics is invasive, there is great interest in developing biomarkers to identify patients with early manifestations of PAH, to assess disease severity, and to predict patients at risk for poor outcomes. While few tests are available clinically, there is an emerging literature suggesting that serum proteins may be useful in this endeavor (Table 1). This section will focus on established and emerging biomarkers for SSc-PH or SSc-PAH.

### 5.1. NT-proBNP and BNP

B-natriuretic peptide (BNP) and its N terminal segment (NT-proBNP) are clinically available tests which have been well-established to be elevated in patients with congestive heart failure of multiple etiologies, but also in PH generally and in SSc-PAH. NT-proBNP, specifically, has been shown to correlate with an increase in right ventricle overload in PAH [38]. Though both showed a correlation, NT-proBNP had a stronger correlation with hemodynamic indicators both before and during the progression of PAH. SSc-PAH incidence has not been shown to be predicted using baseline BNP or NT-proBNP levels, but these markers have been useful in monitoring the severity of disease with elevated levels being associated with higher risk of mortality [39].

### 5.2. Autoantibodies

Among patients with SSc, autoantibody subsets have shown to be highly predictive biomarkers for disease risk stratification, and this is true of PAH as well [40]. A meta-analysis of SSc-PH associated antibodies demonstrated that SSc-PAH patients had a high prevalence of anti-centromere antibodies, anti-U3 RNP antibodies, anti-Th/To antibodies, and antiphospholipid antibodies, while associations were not seen in Scl-70 or RNA polymerase III [40]. Anti-centromere antibodies and antiphospholipid antibodies are both estimated to be present in about half of SSc-PAH patients [40]. The association of SSc-PAH with anti-centromere antibodies, however, may be influenced by survival bias, and recent studies which have controlled for this confounder have not shown an association [41]. An important recent study has also identified a new autoantibody anti-ANPA32a which is present in 4% of SSc patients and portends an increased risk of SSc-PAH [42].

While not clinically available, antibodies against endothelin 1 (ET-1) and Ang receptor type 1 (AT1R) have been identified in patients with SSc and other connective tissue diseases at a much higher rate than in IPAH [43], and have been proposed as both predictive and prognostic biomarkers in SSc-PAH. However, these autoantibodies are not SSc-specific and have also been identified in healthy individuals and in patients with other autoimmune diseases. Both antibodies are thought to contribute to SSc-PAH via increased vascular endothelial reactivity and induction of pulmonary vasculopathy [43]. While SSc derived IgG can mediate AT1R- and ETAR-dependent vasoconstriction, mechanistic studies of anti-ET1 anti-AT1R antibodies have not been able to demonstrate a direct antibody mediated effect and thus whether these antibodies are functional remains to be determined [44].

### 5.3. Proteome-Wide SSc-PAH Biomarkers

A recent proteomic study from the DETECT cohort was performed to identify proteins which may increase in SSc-PAH. This study identified eight proteins (from 313 assessed) which were elevated in SSc-PAH, including RAGE, IGFBP-7, collagen IV, endostatin, MMP-2, IGFBP-2, NT-proBNP, and neuropilin-1, and were validated in an independent cohort [45]. These markers have previously been shown to be relevant in pulmonary vascular remodeling, angiogenesis and cellular growth, and cardiac dysfunction [45]. The combination of these eight proteins was able to discriminate PAH from non-PH in SSc patients.

Another high-throughput proteomic assay of over 1000 proteins identified chemerin and SET (the SET nuclear proto-oncogene) as being associated with SSc-PAH [45]. Chemerin levels were confirmed to be elevated in a replication cohort and to correlate with pulmonary vascular resistance in SSc-PAH patients. Chemerin mRNA was detected in fibroblasts, PA-SMCs/pericytes and mesothelial cells of lung tissue. Moreover, immunofluorescence revealed increased expression of a chemerin receptor, CMKLR1, on SSc-PAH PA-SMC, which is of interest because this receptor can induce PA-SMC proliferation [46].

An older proteomic screen identified midkine and follistatin 3-like (FSTL3) in SSc-PAH with good sensitivity and specificity. The combination of midkine and FSTL3 together was predictive of PAH [47].

### 5.4. Metabolic Biomarkers

A metabolomic study of circulating bioactive lipid molecules found five metabolites distinguished between SSc-PAH and IPAH. SSc-PAH patients had increased levels of fatty acid metabolites, including lignoceric acid and nervonic acid, as well as eicosanoids/oxylipins and sex hormone metabolites [48]. Another recent study found that kynurenine and its ratio to tryptophan (kyn/trp) increased over in patients with SSc who subsequently developed PAH [49]. In both clinical and experimental PAH, higher kynurenine pathway metabolites correlated with adverse pulmonary vascular and RV measurements, and expression of the tryptophan converting enzyme *TDO2* was significantly up-regulated and correlated with pulmonary hypertensive features in tissue.

### 5.5. Cytokines and Chemokines

CXCL4 has been shown to be elevated in SSc generally, and levels are both associated with the development of PAH and poor prognosis amongst PAH patients [50]. SSc-PAH has also been associated with CCL21 levels that have been shown to be elevated in patients with SSc and SSc-PAH, and to be elevated prior to the diagnosis of PAH, and elevated levels were associated with decreased survival [51]. In SSc-PAH patients, IL-32 sera levels were significantly higher when compared with SSc patients without PAH and patients affected by IPAH, and IL-32 sera levels correlated with PA pressures [52]. Another study showed that while cytokine profiles did not differ significantly between SSc patients at high and low risk for PAH, there was evidence of increased levels of VEGF-D in PAH groups patients compared to low-risk PAH groups and HC groups, and cytokines differentiating high-risk PAH patients from low-risk PAH patients were cytokines involved in modulating fibrosis and endothelial cell function including PAI-1, sICAM-1, and BDNF [53].

### 5.6. Additional Candidate Biomarkers

Serum levels of lysyl oxidase (LOX) were elevated in SSc serum and found to inversely correlate with the diffusing capacity of the lung for carbon monoxide diffusing capacity (DLCO). Patients with moderate to severe PAH had higher LOX levels, and lung biopsy specimens showed prominent LOX staining in SSc patients with PAH in the endothelium of remodeled vessels [54].

A recent study found that Pentraxin (PTX-3) levels are significantly elevated in subjects at risk for SSc compared to those at low risk for PH. One explanation for this is the possibility that lower levels of PTX-3 in a patient may lead to an improved ability to regenerate damaged ECs and blood vessels working against the development of PH [55].

Soluble fms-like tyrosine kinase 1 (sFlt-1) and placenta growth factor (PlGF) were elevated in SSc- PH compared to SSc without PH. sFlt-1 positively correlated with right ventricular systolic pressure, and both inversely correlated with DLCO [56].

Micro-RNAs miR-20a-5p and miR-203a-3p were reduced in SSc-PAH and correlated inversely with NT-pro-BNP, and this was particularly prominent in female patients with ACA-positive lcSSc [57].

**Table 1:** Biomarkers of SSc-PAH. For each biomarker or group of biomarkers, the groups compared to SSc-PAH are indicated and the specific outcomes and associations are detailed. Abbreviations: SSc-PAH: systemic sclerosis associated pulmonary arterial hypertension; SSc-no-PAH: SSc not associated with pulmonary arterial hypertension, SSc-AR-PAH: SSc at risk for PAH; IPAH: idiopathic pulmonary arterial hypertension; HC: healthy controls; AUC: area under the curve; HR: Hazard Ratio; PVR: pulmonary vascular resistance.

	Biomarker(s)	Comparison Groups	Association(s)	Reference
Natriuretic Peptides	NT-proBNP	SSc-AR-PAH	Pulmonary Hypertension Severity (mPaP, PVR, Cardiac output, 6MWD, NYHA functional class)	[39]
	BNP	SSc-AR-PAH	Predictors of progression to SSc-PAH from SSc-AR-PAH (BNP: HR (95% CI) 0.6 (0.1–5.7); NT-proBNP: 1.6 (0.2–14.3), composite BNP/NT-proBNP group predicted mortality (HR 3.81 (2.08–6.99), *p* < 0.0001)	[39]
Autoantibodies	Anti-centromere, Anti-U3 RNP, Anti-Th/To, Antiphospholipid	SSc with alterative antibodies	SSc-PAH incidence	[40]
	Anti-ANPA32a	Anti-ANPA32 negative SSc	Echocardiographic evidence of pulmonary hypertension (69% versus 37%; *p* = 0.012)	[42]
	Antibodies against Endothelin 1	SSc no-PAH, IPAH	Active SSc-PAH (SSc-PAH vs. IPAH: ATR1: 68.8/85.5 (0.772) Anti-ETAR: 72.5/85.5 (0.786)) (Non–SSc-PAH vs. SSc-PAH: ATR1: 68.8/78.0 (0.735) Anti-ETAR: 70.0/82.4 (0.754))	[43]
	Ang receptor type 1 (AT1R)	SSc no-PAH, IPAH	Mortality (anti-AT1R: 68.2% and a specificity of 62.2% (AUC = 0.669; *p* = 0.03) and Anti-ETAR antibodies: sensitivity of 68.2% and a specificity of 71.1% (AUC = 0.672; *p* = 0.02).	[43]
Proteome-wide SSc-PAH Biomarkers	RAGE, MMP2, collagen IV, endostatin, neurolipin-1, IGFBP-2, NT-proBNP, IGFBP7	SSc no-PH	AUC 0.741, sensitivity of 65.2% and a specificity of 68.9%	[45]
	Chemerin	SSc-no-PAH, HC	Correlates with PVR (ρ = 0.42, *p* = 0.04)	[46]
Metabolic biomarkers	Nervonic acid, Lignoceric acid, Eicosanoids/oxylipins, Sex hormone metabolites	IPAH, SSc no-PH, SSc-PH	Present in SSc-PAH not in IPAH (85.5% of accuracy (95% CI, 82.8–88.3)	[48]
	Kynurenine, kynurenine to tryphophan ratio	pre-SSc-PAH, SSc no-PAH	Precursor to SSc-PAH, severity of disease, shorter survival times	[49]
Cytokines	CXCL4	SSc no-PAH	Precursor to SSc-PAH, earlier development of pulmonary arterial hypertension as determined on right-heart catheterization (HR 8.33; 95% CI, 4.43 to 15.72; *p* < 0.001)	[50]
	CCL21	iPAH, SSc-PAH, SSc non-PAH, HC	Mortality (HR 2.1, 95% CI 1.21–3.70 [*p* = 0.008])	[51]
	IL-32	SSc non-PAH, iPAH, HC	mPAP and sPAP levels	[52]
	PAI-1, sICAM-1, BDNF, VEGF-D	SSc-High risk for PAH, SSc-Low risk PAH, HC	Profile for patients at high risk for SSc-PAH based on right heart catheterization	[53]
Additional Candidate Biomarkers	Lysyl oxidase (LOX)	Later-SSc, Early-SSc, PRP, HC	Inversely correlated with DLCO	[54]
	Pentraxin (PTX-3)	healthy controls, SSc-PH, SSc-high risk for PH, SSc-low risk PH	High risk for SSc-PAH (High risk: diffusion capacity (DLco) less than 55% with a forced vital capacity (FVC) greater than 70%, an FVC/Dlco ratio of >1.6, or a right ventricular systolic pressure on an echocardiogram greater than or equal to 40 mm Hg)	[55]
	Soluble fms-like tyrosine kinase 1 (sFlt-1), Placenta growth factor (PlGF)	SSc-PH, SSc-no PH	sFlt-1 (*p* = 0.3245; *p* = 0.01) positively correlated with right ventricular systolic pressure. PlGF (*p* = 0.03) and sFlt-1 (*p* = 0.04) positively correlated with the ratio of forced vital capacity to diffusing capacity for carbon monoxide (DLCO), and both inversely correlated with DLCO (*p* = 0.01)	[56]
	Micro-RNAs: miR-20a-5p and miR-203a-3p	lcSSc-ACA, SSc-APAH, SSc-no PAH	Occurrence of SSc-APAH in female patients with ACA-positive lcSSc	[57]

While only BNP/NT-proBNP and autoantibodies are routinely used in SSc-PAH clinically, the emerging body of literature suggests that addition of additional serologic tests which incorporate aspects of altered inflammation, metabolism, and endothelial damage into assessment may be able to improve screening, diagnosis, and monitoring. Future registries and interventional studies in SSc-PH should look to further leverage and validate these emerging biomarkers so that their utility can be better translated to clinical practice.

## 6. PAH Animal Models

While numerous models of PH have been proposed as potential systems to pre-clinically study IPAH and have been extensively reviewed elsewhere [30,58], this review will focus on well-established PH models with severe vascular disease and models which have been proposed to more closely model SSc-PAH or CTD-PAH due to inflammatory and/or systemic features (Table 2).

A. Models of IPAH

### 6.1. Monocrotaline

Injection of monocrotaline (MCT) in rats has been a widely used model used to study PAH due to (i) ease of induction of PH and (ii) severe phenotype including robust pulmonary vascular remodeling, significantly elevated RV/PA pressure, and right ventricle dysfunction [30]. PH is induced through injection with a single subcutaneous or intraperitoneal injection of monocrotaline, making this model appealing due to its simplicity, reproducibility, and low cost [65]. MCT can also be combined with hypoxia or pneumonectomy to model more severe disease with plexiform lesions. However, response to MCT varies across different strains of rats, and is confounded by adverse reactions in other organs as well [30]. MCT induced PH is relatively easily reversible, and therefore does not fully reflect the progressive nature of human PAH [66].

While the exact mechanism of monocrotaline (a plant-derived alkaloid) is unknown, it induces pulmonary artery endothelial cell dysfunction, vasoconstriction, and perivascular inflammatory infiltrate followed by arterial hypertrophy and adventitial fibrosis [30]. MCT causes endothelial cell damage through disruption of endothelial nitric synthase and deregulation of nitric oxide signaling, leading to lung vasculopathy. MCT rats also share other distinctive molecular characteristics with PAH patients, including reduced BMPR2 expression and increased TGF-β expression. Moreover, MCT stimulates an oxidative stress response and apoptosis [67]. Additionally, MCT induces a significant inflammatory cell infiltrate in the pulmonary arteries [68] and genomic studies have shown that TNF/NFkB signaling is the most dysregulated pathway in immune cells and particularly monocytes in this model [69]. There are no features beyond PH which have been suggested to indicate that MCT is a model of scleroderma; these animals to do not have systemic vasculopathy or fibrosis of the skin or lung, and have not been shown to develop autoantibodies.

### 6.2. Sugen/Hypoxia 

The Su/Hx rat model is widely used due to its ability to cause severe and irreversible PH following the prevailing model of PAH development [30]. Sprague Dawley (SD) rats treated with the VEGF receptor antagonist Sugen (Su) 5416 and chronic hypoxia for 3 weeks display pulmonary hypertension, concentric hypertrophy, plexiform lesions, and increases in RV pressure and RV hypertrophy [70]. Mechanistically, due to its inhibition of VEGF, Sugen treatment inhibits proper maintenance, differentiation, and function of endothelial cells, demonstrated by dramatic pulmonary endothelial cell death [71]. At a molecular level, this manifests as loss of endothelial growth signals, inflammation, oxidative stress, and is associated with increases in TGF-β, BMP, Smad, Hif-1α, and MAPK signaling pathways [65]. As in MCT, pneumonectomy has been reported to make the model more severe and more closely represent human disease as removal of one lung leads to increased and turbulent pulmonary blood flow and more prominent vascular remodeling [72]. Sugen/Hypoxia treatment has also been replicated in mice; however, mice develop less severe disease than rats and the phenotype dissipates over time [73]. As in MCT, there are no features beyond PH to suggest that hypoxia or Su/Hx represents a model of scleroderma.

### 6.3. BMPR2 Transgenic/Knockout Animals

As described earlier, BMPR2 is well-established as a genetic risk factor contributing to PAH. Many mouse lines were engineered to investigate the impact of BMPR2 signaling deficiency on PAH development. In particular, mice with endothelial cell-specific heterozygous or homozygous BMPR2 inactivation demonstrate sustained elevation of RV pressure associated with perivascular accumulation of inflammatory cells and occlusion of distal PAs [74]. Several BMPR2 mutant strains which do not spontaneously develop PH have been assessed for increased responsiveness to PH triggers like 5-lipoxygenase exposure, MCT, Sugen, chronic hypoxia, and Su/Hx. However, only over-expression of 5-lipoxygenase produced additional effects on PH development in most, supporting a “two-hit” model of PAH progression in which both BMPR2 deficiency and stressful conditions are critical for the development of PAH [30]. More recently, BMPR2^−/−^ homozygous mice with inducible smooth muscle specific knockout were shown to develop increased PA thickness and elevated RVSP after exposure to hypoxia [75]. The animals phenocopy patients with genetic mutations in BMPR2 leading to PAH but lack autoimmunity, microvascular vasculopathy, or fibrosis and thus should be considered a model of hereditary PAH but not SSc-PAH.

B. Models of Scleroderma or Connective Tissue Disease with Pulmonary Vasculopathy

### 6.4. Fra-2 Transgenic Mice

Fos-related antigen 2 (Fra-2) is a member of activator protein 1 family of transcriptional regulators. Its activation results T cell mediated inflammation and obliterative vasculopathy which precedes dramatic promotion of fibrosis, which is characteristic of SSc [76]. Fra-2 transgenic mice develop vasculopathy which is most prominent in the lungs and is histologically characteristic of PH including intimal thickening with concentric laminar lesions, medial hypertrophy, perivascular inflammation, adventitial fibrosis, and the absence of pulmonary venous occlusion, although the RV pressure in these mice are only mildly elevated [77]. The pulmonary inflammatory lung disease is significant, although this prominently features a T cell component which is somewhat distinct from SSc-ILD which generally has a more macrophage rich infiltrate. The mice do demonstrate skin fibrosis and decreased dermal vessel density consistent with a systemic micro-vasculopathy. By 20 weeks, the mice die due to respiratory insufficiency, primarily from severe fibrosis of the lungs [63]. As the Fra-2 mouse model demonstrates PH associated with development of severe inflammatory-fibrotic lung disease, it likely provides a valuable model to better understand Group 3 PH but does not appear be as reminiscent of PAH (Group 1) pathophysiology. 

### 6.5. Fli-1/Klf5 Mice

Friend leukemia virus integration1 (Fli1) is a repressor of the type 1 collagen gene and is a key regulator of fibrosis, with its deficiency associated with an overproduction of collagen [62]. Fli1 is also down-regulated in idiopathic PAH patients, and it is thought that this may contribute to the pathogenesis of vascular lung complications [78]. Transgenic Fli1 mice with a homozygous deletion of the C-terminal transcriptional activation domain develop elevated fibrosis of the skin and lung due to overproduction of collagen [79]. In addition, endothelial-specific Fli1 knockout mice have been shown to develop arteriolar stenosis, dilation of capillaries, and increased vascular permeability without concomitant fibrosis in both skin and lung [80].

When combined with heterozygosity for Kruppel-like 5 (Klf5), Fli1 heterozygous mice spontaneously develop dermal fibrosis, increased B cell accumulation and collagen deposition in the lung, interstitial pneumonia, and obliteration of precapillary arterioles at 4 months of age and are thus thought to be an especially relevant model for SSc [81]. While the histologic lesions are reminiscent of PH, there has not been specific characterization of features such as RV hypertrophy or measurement of RV pressure reported in these animals. As in the Fra-2 Tg mice, these animals develop significant fibrosis with a prominent lymphocytic cell infiltrate (B cell, rather than T cell in this model) and therefore it is unclear whether they represent a combination of Group 1 and Group 3 PH or are predominantly a model of Group 3 PH.

### 6.6. TNF Transgenic Mice

**Figure 2:**
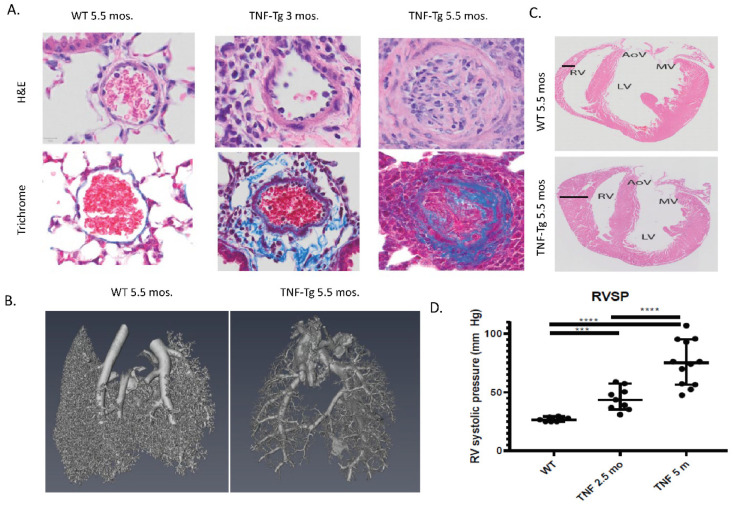
Characterization of the TNF-Tg model of PH. (**A**) Pulmonary vessels (20×, H&E) in TNF-Tg mice demonstrate inflammation at around 3 months, with severe occlusion by 5.5 months. (**B**) μCT demonstrating significant pruning of the distal vasculature in TNF-Tg lungs. (**C**) Heart histology (5X, H&E) reveals significant right ventricular (RV) hypertrophy in TNF-Tg mice at 5.5 months of age. (**D**) Right heart catheterization demonstrates significantly elevated right ventricular pressures (RVSP) in TNF-Tg mice. *** *p* < 0.001, **** *p* < 0.0001.

The tumor necrosis factor-transgenic (TNF-Tg) mouse model is an established model of inflammatory arthritis which has recently been shown to develop severe pulmonary pathology that resembles human CTD-PAH (Figure 2). TNF-Tg mice develop severe obliterative and fibrotic pulmonary vascular lesions as well as progressive right ventricle pressures with female mice dying from cardiopulmonary disease by six months of age [64]. At 3 months, extensive remodeling of adventitial tissue is seen in the lungs followed by cellular infiltration and progressive pulmonary vessel occlusion [64]. 

Lungs of TNF-Tg mice have decreased vessel numbers and μCT scans demonstrate significantly reduced distal pulmonary vasculature. Bone marrow chimera experiments demonstrated that TNF-Tg mice with wild type (WT) bone marrow developed pulmonary hypertension while WT mice receiving TNF-Tg bone marrow did not, indicating that PAH is driven by stromal cell TNF production rather than by hematopoietic cells. The gene expression pattern of TNF-Tg mice was compared to multiple human lung diseases and found to be genomically similar to CTD-PAH [64]. While these mice do demonstrate a significant cellular interstitial infiltrate similar to an inflammatory non-specific interstitial pneumonitis (NSIP) pattern with a prominent myeloid infiltrate, they do not have evidence of lung fibrosis with trichrome staining showing collagen accumulation isolated to the pulmonary vessels [82].

Single-cell RNA sequencing has shown that TNF-Tg mice demonstrate reduced endothelial cell numbers, a loss of pericytes, increased numbers of smooth muscle cells, and a shift in fibroblast phenotype [83]. Pathways over-expressed in TNF-Tg lungs include inflammation, extracellular matrix, angiogenesis, and vascular adhesion. BMPR2 interactions with BMP ligands in TNF-Tg PAH lungs were impaired while there is a maladaptive BMP2 signaling axis [84]. Moreover, these animals show down-regulation of BMPR2, particularly in endothelial cells. Crossing TNF-Tg mice to mice lacking TNFR1 or TNFR2 has shown that the PH is driven by TNFR1 and not TNFR2 signaling [83].

There is no single animal model which recapitulates SSc-PH in its entirety and therefore future studies seeking to study SSc-PH pathophysiology or therapy should either use multiple models with different features to address different aspects of disease or should draw conclusions based on the features in which the specific animal chosen overlaps human disease and point out the limitations of the given model for other features of this complex phenotype. For example, treatment studies looking to demonstrate a reduction in PA pressure will likely want to use a rat model such as MCT or Su/Hx, but to make the findings generalizable to SSc, may want to confirm findings in a model such as the Fra-2 or TNF-Tg. Likewise, studies looking to understand the biology of PH secondary to ILD may want to use the Fra-2 or Fli-1/Kl5 mice, but these studies should not try to generalize their findings to PAH. One avenue which should also be considered moving forward is the development of a humanized model of SSc-PAH which uses primary cells from patients to drive PH development as this may allow for the development of systems which more closely model human disease.

## 7. Cellular Pathogenesis

Because of the lack of studies with access to fresh tissue in SSc-PAH which have been able to evaluate cellular makeup, we here review cellular pathogenesis of PAH generally with an emphasis on cellular processes known to be relevant to SSc (Figure 1).

### 7.1. Endothelial Cells

In healthy tissues, endothelial cells (ECs) line the inner wall of blood vessels and regulate blood flow, vessel permeability, and maintain proper arterial pressures. There is a general loss of endothelial cell function in PAH, indicating a prominent role for ECs in PAH initiation and progression. Many characteristics of PAH are consequences of EC dysfunction, including pulmonary inflammation and coagulation, and a combination of endothelial cell death and proliferation [85]. In particular, PAH leads to a loss of angiogenic potential in microvascular ECs and therefore loss of the microvascular EC network [86]. There is also an increase in adhesion molecules (such as ICAM1 and VCAM1) and inflammatory cytokines in the endothelium of distal PAH arteries, resulting in inflammatory cell recruitment accumulation [87]. Early stages of PAH involve EC dysfunction and apoptosis, while the later stages involve apoptosis-resistant ECs and endothelial senescence.

High shear stress and hypoxia affect endothelial structure and directly contribute to pulmonary vascular remodeling [88]. Shear stress is particularly important in the pathogenesis of PAH as failed EC responses to altered flow causes changes in pulmonary pressures [89]. ECs also become more prone to senescence and apoptosis in response to shear stress.

Endothelial cells in PAH have been shown to have a predilection to undergo mesenchymal cell transition (EndoMT), whereby they undergo transcriptional reprogramming and shift toward a mesenchymal cellular phenotype normally associated with smooth muscle cells (SMCs) and fibroblasts [90]. Hypoxia, dysregulated BMPR2 signaling, inflammation, and oxidative stress all contribute to EndoMT. 

Patients with SSc experience a similar loss of microvascular ECs and EC dysfunction. In particular, defective angiogenesis, defective vasculogenesis, and EndoMT have all been implicated in the pathogenesis of SSc and SSc-PAH [91]. The specific subtypes of endothelial cells which are lost in SSc and the proliferation or persistence of other endothelial cell types is an area which is actively being studied, and analysis of single-cell RNA sequencing studies will likely highlight important endothelial subtypes and gene expression patterns which make SSc-PAH endothelium uniquely susceptible to PH. 

### 7.2. Vascular Smooth Muscle Cell

Vascular smooth muscle cell (VSMC) proliferation is a prominent feature of PAH. In PAH patients, SMCs show excessive proliferation, migration, and invasion compared to in normal conditions [92]. This change in VSMC behavior is called phenotypic switching. VSMCs which at baseline demonstrate a contractile phenotype differentiate into a synthetic phenotype and become massively proliferative and increase their migratory capacities in PAH [93]. This switch is a key event in the early stages of PAH and is a possible therapeutic target. Senescent vascular SMCs are also present and may be key drivers of vascular inflammation and lung destruction and contribute to pulmonary vascular remodeling [93].

To date, there has not been a significant study contrasting SMC phenotypes in SSc-PAH and PAH of other subtypes and future studies may want to determine if there are significant SMC phenotypes that are unique to scleroderma.

### 7.3. Fibroblasts

Fibroblasts are mesenchymal cells that maintain tissue homeostasis and by producing complex extracellular matrix through the production of collagen, fibronectin, and elastin and are present in the adventitial layer of pulmonary vessels [94]. Upon stimulation with factors such as TGF-β, fibroblasts will take on a more contractile myofibroblast phenotype. In PAH patients, myofibroblasts migrate into the adventitia of the arterial wall, resulting in the thickening of the blood vessel and contributing to vascular fibrosis.

This process is considered a critical factor in the pathogenesis of pulmonary vascular remodeling in both SSc and PAH, with SSc patients in particular having an activated fibroblast phenotype and increased myofibroblast differentiation. However, the relative contribution of fibroblasts to the PAH lesion remains relatively poorly characterized, and the emergence of multiple fibroblast phenotypes which are unique to SSc in skin and ILD [95,96,97] suggests that similar analyses in the lungs of SSc-PAH may yield additional insights.

### 7.4. Pericytes

Pericytes are mesenchymal cells that are located in capillary microvasculature that provide structural and biochemical support to endothelial cells. In PAH patients, they can differentiate into myofibroblasts, leading to increased collagen production and driving tissue fibrosis [98]. Pulmonary pericytes were revealed to trigger progression of PH vascular changes in blood vessels and may represent an underappreciated contributor to the pathogenesis of disease [99]. However, these cells are difficult to study and there is relatively little characterization of the role of pericytes in SSc-PH.

### 7.5. Myeloid Cells

Macrophages are the predominant inflammatory cells infiltrating around PAH lesions and are an important factor in the progression of pulmonary vascular remodeling and contribute to disease pathogenesis by secreting cytokines, chemokines, and growth factors [100]. An imbalance in macrophages differentiation into M1 or M2 phenotypes has been implicated in PAH [101]. Macrophages and VSMCs express similar cytokines that, in a PAH disease environment, can stimulate each other and aggravate VSMC proliferation [102]. Immature dendritic cells have also been found to accumulate in remodeled pulmonary vessels and may play a role as antigen presenting cells in PAH [103].

There is a rich literature suggesting a pathogenic role for myeloid cells in SSc, including in skin and lung fibrosis [104,105]. However, phenotypic characterization of myeloid cells in SSc-PAH has been more limited mostly to studies of peripheral blood gene expression. Characterization of tissue-resident macrophages, therefore, represents an important opportunity to better understand the drivers of inflammation in SSc-PAH.

### 7.6. Lymphocytes

Mounting evidence suggests that immune dysfunction can explain both the accumulation of inflammatory cells and the overabundance of cytokines and chemokines in PAH patients. The infiltration of immune cells and lymphocyte proliferation both contribute to pulmonary vascular wall thickening. Both Th1 and Th2 driven inflammation has been reported in PAH. During the Th1/Th17 response, vessels are invaded by cytotoxic T cells, autoreactive B cells, autoantibodies, and activated macrophages. These cells produce TNF-α and IL-6, which can continue to promote vascular remodeling [106]. During the Th2 responses, eosinophils and mast cells are recruited and infiltrate the vessel wall, and TGF-β signaling drives cytokines like IL-4 and IL-13 to promote more fibrotic responses.

Increases in CD8+ cytotoxic T cells are also found at higher levels in PAH patients and may be associated with pulmonary arterial calcification [107]. Meanwhile, Treg cells, which have anti-inflammatory properties, have been found to be decreased in PAH lungs [108].

While the role of B cells in PAH is less clear, B cell activation markers are elevated in SSc-PAH [109]. While none have been shown to be directly pathogenic, autoantibodies are thought to play a role in disease with antibodies targeting antinuclear antigens, endothelial cells, and fibroblasts being found in both idiopathic and systemic-sclerosis-associated PAH [110,111].

The cellular pathogenesis underlying difference between SSc-PAH and other forms of PAH remains to be fully elucidated. Given the pathologic features including increased venous involvement and decreased plexogenic lesions, it is likely that different subsets of endothelial cells become activated, apoptotic, and undergo endothelial–mesenchymal transition in different subsets of PAH. Likewise, given the emergence of fibroblast sub-populations which are seen specifically in scleroderma skin and lung disease [27,95,96,97], it is likely that there may be SSc-PAH specific fibroblast, pericyte, and/or smooth muscle cell populations. More detailed studies using single-cell and spatial omic approaches should hopefully lead to better clarification of these differences.

## 8. Intracellular Signaling

Although underlying molecular mechanisms in SSc-PAH include activation of inflammatory and fibrogenic pathways, the molecular pathogenesis of SSc-PAH is complex and not fully elucidated. Given that SSc-PAH shares many molecular phenotypes with both SSc and PAH, we will highlight key signaling pathways identified in SSc and PAH with emphasis on promoting understanding of the molecular pathogenesis of SSc-PAH.

### 8.1. BMP Signaling Pathway

The bone morphogenetic protein (BMP) signaling pathway, especially BMPR2, has long been implicated in PAH with loss-of-function and mutation of BMPR2 function induced by genetic or acquired mechanisms [112]. BMPR2 is a serine/threonine kinase transmembrane protein belonging to the TGF-β receptor superfamily. The classical BMP signaling pathway is activated upon binding of BMP ligands to BMPR2, forming complex by recruiting and phosphorylating ALK1, 2, 3, or 6, then subsequently phosphorylating receptor-regulated Smads (R-Smads) including Smad1/5/8. Theses R-Smads form complexes with co-Smads, translocate to the nucleus, and bind to BMP response elements leading to transcriptional regulation of gene expression [113].

Decreased BMPR2 expression was found in SSc-PAH patients as well as a TGF-β-dependent SSc-PAH mouse model [114], and TGF-β impairs BMP signaling via increased proteasomal degradation of BMPR2 [114]. In SSc, BMP7 can block TGF-β-induced EndoMT in both endothelial cells and an SSc mouse model through the Akt/mTOR signaling pathway [115]. Collectively, these findings suggest that the BMPR2 signaling pathway may play an important role in SSc-PAH.

### 8.2. TGF-β Signaling Pathways

TGF-β signaling is well-established as a key driver of both SSc and PAH and is likely to be an important link between the two conditions. TGF-β superfamily members are pleiotropic cytokines that have been reported to mediate fibrosis, inflammation, and vascular biology in human health and disease [116]. The TGF-β pathway is initiated by forming a heterotetrameric complex between TGF-β receptors (TGFβRI and TGFβRII) and ligands. The heterotetrameric complex subsequently leads to autophosphorylation of TGFβRI and TGFβRII regulating the docking and phosphorylation of Smad2/3, which in turn interacts with Smad4 to create a transcriptional complex that translocates to the nucleus and up-regulates or down-regulates the expression of multiple target genes. However, regulatory Smad proteins vary depending on cell type and cell state and can lead to variant Smad-mediated transcription. In pulmonary blood vessels, activated Smad2/3 signaling contributes to proliferation of smooth muscle cells and endothelial cells. However, Smad1/5/8 signaling inhibits this proliferation. Roles of TGF-β signaling pathway in PAH and SSc have been extensively reviewed elsewhere and a full description is beyond the scope of this article [112,117]. Sotatercept, a novel activin/inhibin-targeting drug, showed that restoration of altered TGF-β signaling can improve outcomes in PAH, and represents an exciting novel therapeutic target [118].

In SSc, TGF-β-regulated genes are differentially expressed in organ fibrosis, and are associated with more severe vascular disease [117]. Compared to healthy controls, TGF-β1 levels are significantly decreased in SSc patients. Moreover, levels of TGF-β1 are decreased in SSc-PAH as compared to SSc. Increased levels of TGF-β2 and TGF-β 3 were observed in both SSc and SSc-PAH patients, but SSc-PAH had a higher level of TGF-β2 and TGF-β3 compared to SSc [119] Levy et al. found that miR-26 and miR-let-7d levels were significantly decreased in SSc-PAH versus SSc without PAH and bioinformatic analysis showed that both miRNAs-regulated genes belong to TGF-β signaling pathway or Smad binding gene sets [120]. Although mutations of TGF-β receptor genes contribute to idiopathic and familial PAH, there is lack of association between TGF-β receptor gene polymorphisms and SSc-PAH.

### 8.3. Vasodilatory Pathways

There are three primary vasodilatory pathways of interest in PAH: the nitric oxide (NO) pathway, endothelin (ET) pathway, and prostacyclin pathway. To date, drugs for SSc-PAH primarily act through these three pathways, and drugs targeting these three pathways have been shown to relieve PAH-associated symptoms [1,121]. In PH, pulmonary arterial endothelial cells (PAECs) regulate the contractile and diastolic function of vessels by secreting contractile factors such as thromboxane A2 (TXA_2_) and endothelin-1 (ET-1) as well as diastolic factors such as PGI_2_ and nitric oxide (NO) [122].

#### 8.3.1. Nitric Oxide Pathway

Nitric oxide (NO), a potent vasodilator, is synthesized from the conversion of L-arginine to citrulline by the NO synthase [123,124]. NO promotes vasodilation by increased NO/cGMP/PKG signaling. This pathway is also responsible for catalyzing cGMP production by activating sGC, and subsequently activates cGMP dependent kinase or PKG. The up-regulated NO/cGMP/PKG signaling decreases Ca^2+^ release from the intracellular store leading to SMC vasorelaxation [125,126].

In SSc-PAH patients, NO-synthase is decreased, which leads to less production of NO resulting in vasoconstriction of pulmonary arteries [127]. Moreover, exhaled NO is lower amongst SSc patients with pulmonary hypertension compared to SSc patients without PH [128].

#### 8.3.2. Endothelin Pathway

Endothelin (ET) is a vasoconstrictor produced primarily by endothelial cells with three isoforms: ET-1, ET-2, and ET-3 [129]. Expression of ETs is induced and mediated by TGF-β signaling as well as transcriptional factors such as activator protein 1 (Ap-1), nuclear factor kappa B (NF-κB), forkhead-box protein O1 (FOXO1), HIF-1α, and GATA2 [130]. There are two endothelin receptors: ETAR and ETBR. ETBR can bind to ETs equally, but ETAR shows higher affinity of ET-1 [129].

ET-1 has been found to be up-regulated in SSc and is correlates with severity of PAH in multiple studies, and its expression is elevated in pulmonary arterial endothelial cells [131]. ET-1 also plays critical roles in inducing collagen production and differentiation of fibroblasts into myofibroblast, leading to vascular impairment in SSc [132].

#### 8.3.3. Prostacyclin Pathway

Thromboxane A2 (TXA_2_) and prostacyclin (PGI_2_) belong to the prostaglandin (PG) family [133]. PGI_2_ is secreted from endothelial cells and is a powerful vasodilator and anti-proliferative agent. Under hypoxic conditions, pulmonary vascular smooth muscle cells produce the enzyme cyclooxygenase-2 (COX-2). COX-2, in turn, catalyzes formation of PGI_2_ from arachidonic acid [134]. Upon binding to its receptor, PGI_2_ can increase cyclic adenosine monophosphate (cAMP) levels and decrease the intracellular calcium concentration leading to vascular smooth muscle cell relaxation, whereas in endothelial cells it has a vasoconstricting effect [122]. Previous evidence showed that PAH patients had reduced PGI_2_ synthase and decreased expression of PGI_2_ receptors in the lungs [135]. PGI_2_ was reported to have a long-term effect on promoting angiogenesis.

In SSc-PAH patients, prostacyclin synthase is decreased, leading to a lower level of prostacyclin and increased formation of TXA_2_, contributing to vasoconstriction, thrombosis, and proliferation [136,137]. Taken together, vasoconstriction in SSc-PAH is thought to result from over-expression of TXA2 and ET-1 and under-expression of nitric oxide and prostacyclin.

### 8.4. Notch Pathway

The Notch signaling pathway plays critical roles in development, repair, and regeneration of lungs [138]. The Notch pathway is composed of four receptors and five ligands (DLL-1,3,4 and JAG-1,2). Upon ligand binding, Notch receptors are subjected to cleavage and catalyzed by metalloproteinases, causing the translocation of Notch receptor’s intracellular domain to the nucleus, and triggering expression of target genes [139].

Increased expression of Notch1, Notch3, Jagged1, and Herp2 was documented in mouse model of hypoxia-induced PAH and rat model of monocrotaline-induced PH, and soluble Jagged1 treatment can improve the survival of PH by attenuating Notch pathway [140]. A previous study found that SMC-EC contact can initiate activation of the BMPR2-Notch1 pathway, driving EC proliferation and endothelial regeneration [141]. Notch2 promotes endothelial apoptosis and its expression increases in PAECs exposed to hypoxia, TGF-β, and ET-1 [142]. In PAH patients, elevated activation of Notch3 was observed in pulmonary artery smooth muscle cells and Notch3 knock-out mice fail to develop PH under chronic hypoxic conditions [143]. By using an EC-specific progeroid mouse model, a recent study found the over-expression of Notch ligands was observed in senescent ECs and that the Notch pathway is activated by senescent ECs. This study also showed that EC-specific progeroid mice develop worse PH and that Notch inhibition improved the outcome of PH [144]. Inhibiting Notch signaling pathway using the γ-secretase inhibitor dibenzazepine blocked proliferation and migration of PAECs [145]. Collectively, Notch pathways are closely involved in PAH development.

In SSc, over-expression of JAG-1 was reported in skin, and subsequently caused an accumulation of Notch receptor intracellular domain in SSc fibroblasts and collagen release [146]. Notch4 genetic polymorphisms were reported to contribute to SSc susceptibility via promoting endothelial to mesenchymal transition [147]. Differentiation of myofibroblasts from resting fibroblasts was observed in normal fibroblasts by stimulation with recombinant JAG-1-Fc chimeric antibodies, and led to the increased expression of collagens I III and alpha smooth muscle actin, and decreased ECM-degrading enzymes [146]. ADAM17, an activator of Notch pathway, down-regulated the development of skin and lung fibrosis in the HOCL-induced scleroderma mouse model [148]. In a bleomycin-induced model, inhibition of the Notch pathway by DAPT (a γ-secretase inhibitor) resulted in anti-fibrotic effects [149]. Another study showed that the microRNA miR-16-5 inhibits Notch2 signaling-induced myofibroblast activation [150]. Collectively, the Notch pathway up-regulates myofibroblast activation in SSc. Moreover, activation of the Notch pathway in the endothelium leads to EMT [151]. However, study of Notch in SSc-PAH has not been documented, and whether the particular Notch ligands or signaling differ between SSc-PAH and idiopathic PAH or other forms of PH requires further investigation. Considering the roles of Notch pathway in PAH and SSc, it is clear that the Notch signaling pathway affects the pathogenesis of SSc-PAH, but future studies can better clarify whether there is a unique Notch signaling axis that is SSc-specific.

### 8.5. HIF Pathway

Hypoxia-inducible factor (HIF) is a master transcriptional factor for oxygen homeostasis [152]. HIF family members are composed of oxygen-sensitive α subunits (HIF-1α, HIF-2α, HIF-3α) and oxygen-insensitive β subunits (HIF-1β, HIF-2β, HIF-3β). Under hypoxic condition, HIF-α hydroxylation is blocked, and subsequently cause stabilization of HIF-α subunits, which results in binding of HIF-α subunit with HIF-β subunit and nuclear translocation. Activated HIF pathway is responsible for the regulation of vascular tone, angiogenesis, cellular metabolism, proliferation, survival, and autophagic response [152].

In VSMCs, HIF-1α functions as a pathogenic determinant of muscle cell expansion and pulmonary vascular remodeling and contributes to the prevention of PH development. In PAECs, HIF-1α regulates vascular endothelial growth factor (VEGF) by promoting angiogenesis and regulation of EC proliferation. HIF-1α also induces IL-33 expression in lung tissues of mice and patients with PH. When interacting with its receptor Suppression of Tumorigenicity 2 (ST2), IL-33 modulates proliferation, adhesion, and angiogenesis and contributes to PH development [153]. Furthermore, hypoxia enhances HIF-2α stability, which causes increased arginase expression and dysregulates normal vascular NO homeostasis [154]. HIF-2α also interacts with p53 to accelerate EC apoptosis under hypoxia, leading to dysfunction of pulmonary arterial endothelium [155]. Targeting the CD146-HIF-1α axis in PASMCs disrupts vascular remodeling and results in attenuation of PH [156]. The HIF-2-specific inhibitor, PT2567, reduced mean pulmonary artery pressures, right ventricle remodeling, and pulmonary artery wall remodeling responses in hypoxic rats [157].

In SSc patients, blood plasma levels of HIF-1α were significantly increased compared to controls [158]. HIF-1α/VEGF signaling pathway plays an important role in mediating the effect of hypoxia-induced EndMT, in the skin microvascular remodeling of SSc [159]. By analyzing differential transcriptomic data from fibroblasts with and without hypoxia treatment, He et al. identified nine hypoxia-associated hub genes affecting the pentose phosphate pathway, oxidative stress, and lipolysis, which implicated roles of hypoxia-mediated-non-fibrosis mechanisms on SSc pathogenesis [160]. In SSc-PAH patients, a cohort study found that the single nucleotide polymorphism of *HIF1A* gene (rs12434438 SNP) may be associated with severe PAH among SSc patients [161]. While these insights clearly point to a role of HIFs in SSc-PAH, the degree to which hypoxia drives different HIF signaling in SSc patients with and without ILD, for example, has not been studied and may be a relevant consideration for the relative importance/activation of this pathway between patients with Group 1 and Group 3 PH.

Taken together, there is an increasing literature suggesting that most of the key canonical pathways implicated in PAH are also altered in SSc and many have also been linked to SSc-PAH. However, the degree to which each of these pathways is active in the pulmonary artery microenvironment and the differences in signal transduction between SSc-PAH and other forms of PH remain incompletely characterized and represents an area ripe for investigation. Better understanding of the key differences between SSc-PAH and idiopathic and other forms of PH may not only give better insight into why SSc-PAH patients have poorer prognosis, but also open up new treatment avenues that are SSc-PAH-specific.

## 9. Drug Therapy of PAH and SSc-PAH

In this section, we will focus on existing and emerging therapeutic strategies for PAH with an emphasis on those relevant to SSc and SSc-PAH. While additional therapies are indicated for other WHO classes of PH (for example, diuretics in Group 2 PH and surgical thromboendarterectomy for Group 4), these are beyond the scope of this review, which will focus on therapies targeting the molecular abnormalities present in SSc-PAH. In some cases, we will also highlight therapies which have demonstrated benefit or potential benefit for digital ulcerations, which is a common comorbidity in SSc patients with PAH and likely represents a spectrum of the same vascular disease.

### 9.1. Endothelin Receptor Antagonists

Endothelin receptor antagonists (ERAs) target the endothelin pathway by blocking ETA and ETB and help with both vasodilation and antiproliferation. Patients with SSc experience increased levels of endothelin-1 due to endothelial cell injury [162], so ERAs have been suggested as a possible treatment for SSc vascular manifestations including digital ulcers and PAH. Clinically proven ERAs for PAH consist of bosentan, ambrisentan, and macitentan.

Ambrisentan is a selective ETA receptor antagonist. In a randomized clinical trial of patients with idiopathic PAH, patients taking ambrisentan improved time to clinical worsening and 6-min walk distance [163]. If taken in combination with tadalafil, there was a higher percentage of patients with a satisfactory clinical response and an even greater improvement in the 6-min walk distance [163]. Ambrisentan also delayed disease progression and clinical worsening in PAH patients [164]. In SSc, ambrisentan has been used as part of combination therapy upfront with tadalafil and this combination has led to significantly improved outcomes including hemodynamics, RV structure and function, and functional status [165].

Bosentan is a nonselective ETA/B receptor antagonist that blocks ET-1 function. In a double-blind, randomized, placebo-controlled trial, the bosentan treatment group demonstrated improved 6-min walk distance and cardiopulmonary hemodynamics [166], demonstrating its therapeutic potential in the management of PAH. In two randomized, placebo-controlled studies using bosentan for ulcers, it was also shown to prevent new digital ulcers in patients with SSc, although existing ulcers did not show evidence of healing [167]. In another study, Bosetan did not improve the frequency, duration, pain, or severity of Raynaud’s phenomenon, but did significantly improved the functional scores compared to placebo [168]. While no prospective studies have demonstrated efficacy in SSc-PAH, a retrospective study of patients on bosentan for ulcers revealed a significant decrease in PAH incidence [169].

Macitentan is a nonselective ETA/B receptor antagonist that has greater tissue penetration and receptor affinity than bosentan or ambrisentan. Macitentan significantly reduced the risk of PAH-related events or all-cause mortality by 45% compared with placebo [170], which is more efficacious than the other endothelin receptor antagonists, and has shown significant clinical efficacy in IPAH; registry-based data suggest similar efficacy in SSc-PAH, although formal trials are lacking. Macitentan did not, however, demonstrate efficacy for SSc-related digital ulcers in two large randomized clinical trials [171].

### 9.2. Nitric Oxide: Phosphodiesterase Inhibitors and Guanylate Cyclase Agonists

Nitric oxide (NO) is a critical vasodilator that mediates cGMP synthesis, resulting in decreased intracellular calcium concentrations that relaxes muscles [172]. This pathway is regulated through two major drugs: PDE5 inhibitors and guanylate cyclase agonists. PDE5 degrades cGMP and is an important regulator of vasodilation. In patients with PAH, there is reduced NO synthesis and therefore reduced levels of cGMP. PDE5 inhibitors, such as sildenafil, tadalafil, and vardenafil, thus increase cGMP concentrations.

Sildenafil was shown to significantly improve hemodynamics, measured through a reduced mean pulmonary artery pressure, and increased 6-min walking distance in IPAH [173]. While only case series and small studies have been done to evaluate sildenafil in SSc-PAH, it appears to be effective in this setting [174], and a prospective study evaluating it in early disease in currently underway.

PDE-5 inhibitors also have promise in treatment digital ulcers in SSc, as this class of medication is widely used for various forms of constrictive vasculopathies. Despite evidence of efficacy in the clinic, a randomized controlled trial of sildenafil failed to meet its primary endpoint of healing of ischemic digital ulcers, due in part to an unexpectedly high healing rate in the placebo arm, but did show a decrease in the number of ulcers in the sildenafil group at 8 and 12 weeks [175]. 

Tadalafil, a longer-acting PDE5 inhibitor, was demonstrated to significantly improve 6-min walk distance, delay time to clinical worsening, and improve measures of health-related quality of life assessments in IPAH [163]. These improvements were maintained through an extended 1-year observation period [172]. Combination therapy with ambrisentan and tadalafil has shown to be highly efficacious [165] in SSc-PAH, and this combination therapy is considered first-line for patients with SSc-PAH and likely reflects a major reason for improvements in SSc-PAH outcomes in the last 10 years [16].

Vardenafil has also been shown to significantly improve the 6-min walking distance that was maintained for 24 weeks as well as decrease mean pulmonary arterial pressure and pulmonary vascular resistance [176], but has not specifically been studied in SSc.

Guanylate cyclase agonists such as riociguat work on soluble guanylate cyclase and increase cGMP concentrations, relax pulmonary vessels, and have antiproliferative properties [172]. In a phase 3 double-blind study in patients with IPAH, the group receiving riocigut increased their 6-min walk distance, demonstrated significant improvements in pulmonary vascular resistance, and had improved time to clinical worsening [177]. There have not been studies of riociguat in SSc-PAH, but it has been studied for diffuse cutaneous systemic sclerosis skin disease, where it did not demonstrate efficacy, likely due to its failure to reach therapeutic levels in skin [178,179].

### 9.3. Prostacyclin Agonists and Receptor Agonists

Prostacyclin is a vasodilator that promotes cyclic adenosine monophosphate (cAMP) production and inhibits pulmonary artery smooth muscle cell proliferation. Prostacyclin levels are greatly reduced in PAH patients and treatments such as prostacyclin analogs and prostacyclin receptor agonists can be used to restore prostacyclin functions [172]. Prostacyclin analogs include epoprostenol, iloprost, treprostinil, and beraprost, while selexipag is a prostacyclin receptor agonist.

Epoprostenol, treprostinil, and iloprost have been approved for the treatment of SSc-PAH. Intravenous epoprostenol has long been used in PAH and has shown benefits including improved exercise tolerance, hemodynamic parameters, and long-term survival [180]. It has good efficacy in all functional classes of PAH and is a candidate for use in combination therapy for patients in class III or IV heart failure [181]. However, side effects including infections and sepsis, headache, hypotension, nausea, and diarrhea limit the use of IV prostacyclin analogs [172]. Moreover, the need for continuous infusion, careful catheter care, and daily preparation of these medications can be challenging for SSc patients with significant hand impairments. 

Iloprost exists in both inhaled and intravenous form. Inhaled iloprost has a rapid onset of action and can be useful for acute vasoreactivity testing and critical in a PAH crisis [172]. A study found that a majority of SSc patients with digital ulcers who received monthly IV iloprost treatment resolved their digital ulcers [182], suggesting that this class may also have benefit for ulcers and peripheral vascular complications of SSc.

Treprostinil can be administered intravenously, subcutaneously, inhaled, and orally. Intravenous and subcutaneous treprostinil dose-dependently increase exercise tolerance and improve symptoms, quality of life, and hemodynamic parameters [183]. Inhaled treprostinil was shown to improve exercise capacity as measured via an increased 6-min walk distance [184], and pulmonary function as measured via forced vital capacity [185]. In a trial of SSc-ILD patients treated with inhaled treprostinil, PH (Group 3 PH) demonstrated benefit and suggests that this may be a useful strategy in patients with combined SSc-ILD and SSc-PAH [184]. Current treatments of patients with group 3 PH often include systemic pulmonary vasodilators which have only been approved for group 1 PH and carry a risk of ventilation–perfusion mismatching. Inhaled treprostinil promises to be an effective treatment for patients with group 3 PH without this risk through redirection of blood flow to more ventilated areas of the lung. Moreover, this suggests that pulmonary vascular abnormalities in SSc-ILD may be amenable to vasodilatory therapy, and that rather than focusing only on immunomodulation and anti-fibrotic therapy, as is typical in SSc-ILD, targeting the vasculature may also derive additional benefit in this population, especially given emerging data about endothelial cell cross-talk with fibroblasts and immune cells in interstitial lung disease [186].

Selexipag is a prostacyclin receptor agonist with high affinity for the prostacyclin receptor, leading to a reduction in adverse reactions compared to prostacyclin analogs [172] Selexipag has been shown to reduce the risk of death or PAH-related complications [187] in IPAH. In a study of CTD-PAH patients, selexipag delayed reduction in functional capacity or need for additional therapy, and was well-tolerated [188].

There have not been prospective studies of patients with severe Raynaud’s/digital ulcers and the subsequent development/severity of SSc-PAH, but given the efficacy of endothelin receptor antagonists and phosphodiesterase inhibitors for both manifestations, it is reasonable to posit that initiation of these therapies in patients with Raynaud’s and significant risk for PAH (which could be stratified based on clinical features) may be able to prevent or blunt PAH. Alternatively, it is possible, as has been suggested with ACE inhibitors in scleroderma renal crisis [189], that early initiation or therapy may make it harder to make an early diagnosis of SSc-PAH and could delay other appropriate advanced therapies. Trials looking at early initiation would therefore be important to understand if these drugs may be disease-modifying and should be started earlier rather than waiting for evidence of damage.

### 9.4. Immunomodulation

Dysregulated immune responses contribute to vascular remodeling and proliferation of PAH. Treatments that allow for regulation of immune response may therefore have promise in treating PAH. Rituximab, an anti-CD20 monoclonal antibody which results in B-cell depletion, showed promise as a treatment for SSc-PAH as it improved exercise tolerance, but the study did not reach its primary endpoints of improved 6-minute walk distance [190]. Tocilizumab is an anti-IL-6 agent that is currently being evaluated on the basis of several animal studies that suggest IL-6 receptor upregulation on vascular smooth muscle cells as a contributor to PAH symptoms [191]. Given the complex immunopathology of SSc-PAH, there are many additional pathways including TNF, interferon, and IL-13 which may be considered in future studies. 

Despite the clear role of immune dyregulation in SSc-PAH, study design will be important in order to show a benefit of immune-mediated therapies, especially given that combination vasodilator therapies are the standard of care. The recent clinical trial of rituximab [190], for example, recruited patients with largely later-stage and refractory PAH, which is less likely to respond to immunomodulation, and only a subset of patients demonstrated a response. Future studies will likely need to add immunomodulation to standard-of-care combination vasodilation (with endothelin receptor antagonists and phosphodiesterase inhibitors), but should likely be randomized to be added at diagnosis/treatment initiation rather than only added in patients who fail initial therapy. Another important consideration is molecular phenotyping to improve the likelihood of benefit. Trials may want to enrich patients with established biomarkers (BNP, etc.), cellular biomarkers (flow-cytometry based), or molecular signatures (gene expression in PBMCs to demonstrate target engagement) to increase the likelihood that these studies will show clinical benefit.

### 9.5. Iron Supplementation and Anticoagulation

To help regulate hypoxic stress in PAH patients, iron supplementation has been proposed as a possible way to improve outcomes in PAH since iron availability affects the response to hypoxia. However, a phase 2 clinical trial evaluating intravenous iron did not show improvements in hemodynamic or functional parameters at 12 weeks [191]. However, intravenous iron helps improve symptoms of dyspnea and quality of life, especially in SSc-PAH patients who have to iron deficiency due to recurrent gastrointestinal (GI) hemorrhaging in the setting of GI vascular malformations (AVMs) and can be used adjunctively in such settings. Of note, one therapy which is part of the treatment algorithm for IPAH which is considered relatively contra-indicated for SSc-PAH is warfarin. While the antithrombotic role has demonstrated benefit, likely due to prevention of microthrombi in other forms of PH, the lack of evidence to support its use [192] and frequent multifactorial anemia and risk of bleeding in SSc-PAH has led to an expert consensus that warfarin should be avoided in SSc-PAH.

### 9.6. Sotatercept

Pulmonary vascular remodeling in PAH is driven primarily by unchecked proliferation and reduced apoptosis of endothelial cells. This is caused in part due to altered signal transduction of the TGF-β superfamily, which includes BMPR2 and ActRIIA [193]. Sotatercept is a ligand trap that restores BMP signaling and reduces TGF-β signaling. In a recent trial, sotatercept showed significant improvement in pulmonary vascular resistance and pulmonary artery pressure in PAH patients [193]. It also showed reduction in pulmonary vascular resistance in patients receiving active monotherapy, double therapy, or triple therapy [118]. In a seminal trial, sotatercept has now been shown to decrease the risk of death or nonfatal clinical worsening events by 84% compared to placebo in PAH [193], and is therefore considered an agent with very high clinical upside, and has recently earned FDA approval for PAH. Only a small number of SSc patients were included in the phase III trial, however, and further studies in this population are needed, especially as bleeding GI vascular lesions were a side effect and SSc patients already have an increased risk of GI bleeding.

### 9.7. Combination Therapy

Early initiation of combination therapy with ET receptor antagonists and PDE5 inhibitors for PAH and SSc-PAH have markedly improved treatment response [6]. Due to the multifactorial pathogenesis of SSc-PAH, it is likely that additional targets for combination therapy may also be warranted, and the future of combination therapy may include one or more vasodilators, a therapy targeting TGF-beta signaling, and an immunomodulator. Given the relatively small population of patients with SSc-PAH and therefore the difficulty in recruiting trials in this disease, in addition to randomized controlled clinical trials, large registry-based studies which compare combination approaches in real-world settings will also be important to get a sense of both efficacy and safety of combinatorial approaches. Moreover, given that vasodilatory medication is generally prescribed by pulmonologists while immunomodulatory medication is generally prescribed by rheumatologists, it is imperative that these combination treatment decisions be made in a multidisciplinary way in clinical settings.

## 10. Conclusions

Pulmonary hypertension is a frequent and severe comorbidity of SSc. While PAH is the most common form of PH in SSc, scleroderma patients are at risk for all WHO groups of PH, and therefore careful screening regimens and clinical phenotyping are necessary. While SSc-PAH shares many pathologic features and signaling pathways with other forms of PAH, SSc patients have a number of unique risk factors and comorbidities which make this a unique form of systemic vasculopathy which requires expert referral for diagnosis and treatment. Recent advances in the understanding of PH biology which have arisen from both animal and human studies have yielded significant new insights into the cellular and molecular mechanisms underlying this devastating disease, identified putative biomarkers, and helped create a series of novel therapeutics.

Over the past decade, outcomes for patients with SSc-associated PH have improved, particularly among those with Group 1 PH (PAH). Several factors have likely contributed to better outcomes including better screening and better clinical management of patients with SSc-PAH with more treatment options including more widespread use of early combination therapy. However, there is still a need for more efficient screening to detect PAH in SSc patients with asymptomatic disease and better treatment regimens, particularly for patients with WHO Group 2 and 3 diseases.

Even with modern therapy, this disease is complex, and outcomes remain poor compared to SSc patients as a whole. Therefore, new approaches to diagnosis and treatment are needed. Given the significant role of the immune system and TGF-beta signaling in SSc, the potential for combining immunomodulation or TGF-β targeted therapies with vasodilatory therapy holds promise as a future therapeutic avenue.

## Figures and Tables

**Table 2:** Animal models of pulmonary hypertension. Abbreviations: PH: Pulmonary hypertension; ®: Rat; (M): Mouse; PA: Pulmonary Artery; N.R.: not reported; RV: right ventricle.

PH Animal Model	Vessel Occlusion	Elevation of PA Pressure	RV Remodeling	Interstitial Lung Disease	Perivascular Inflammation	Spontaneous Regression	Systemic Features	Plexiform Lesions	Reference
Monocrotaline ®	+++	++++	+++	−	+++	+	−	+++	[59]
Sugen/Hypoxia ®	++++	++++	+++	−	+++	−	emphysema	+++	[60]
Sugen/Hypoxia (M)	++	++	++	−	++	+	−	−	[30]
BMPR2+/− (M)	++	+	++	−	++	−	−	+	[61]
Fli-1/Klf5 het. (M)	+	n.r.	n.r.	fibrotic	++	−	skin fibrosis	−	[62]
Fra-2 (M)	++	+	++	fibrotic	++	−	skin fibrosis	−	[63]
TNF-Tg (M)	++++	+++	+++	inflammatory	++++	−	arthritis	+	[64]
